# Systematic evaluation of TP53 codon 72 polymorphism associated with onset and progression of oral potentially malignant disorders

**DOI:** 10.1186/s12903-023-03316-0

**Published:** 2023-09-12

**Authors:** Huangkai Li, Yu Liu, Shanxin Zhou, Qin Zhou, Xi Yang

**Affiliations:** 1 School of Stomatology, Hainan Medical College, Haikou, 571199 China; 2 Department of Oral and Maxillofacial-Head and Neck Oncology, Shanghai Ninth People’s Hospital, Shanghai Jiao Tong University School of Medicine, Shanghai, 200011 China; 3 College of Stomatology, Shanghai Jiao Tong University, National Center for Stomatology, National Clinical Research Center for Oral Diseases, Shanghai Key Laboratory of Stomatology, Shanghai Research Institute of Stomatology, Shanghai, 200011 China; 4 Department of Oral Surgery, Shanghai Ninth People’s Hospital, Shanghai Jiao Tong University School of Medicine, Shanghai, 200011 China

**Keywords:** Oral potentially malignant disorders, Oral cancer, TP53, Meta-analysis, Single-nucleotide polymorphisms

## Abstract

**Background:**

Recently, a systematic review and meta-analysis demonstrated that overexpression of p53 immunoprotein was significantly associated with progression risk of oral potentially malignant disorders (OPMD). However, the results of investigations on TP53 genetic typing in OPMD were inconsistent and inconclusive.

**Methods:**

A systematic evaluation was conducted to identify all eligible case–control studies on the association of TP53 codon 72 polymorphism with both onset and progression of OPMD.

**Results:**

A total of 768 OPMD patients and 1173 healthy individuals were identified from 12 eligible case–control studies on TP53 codon 72 polymorphism OPMD onset. In overall and subgroup analyses, no significantly risk of OPMD onset was observed in the cases for genetic models including allele C vs. G, homozygote CC vs. GG, heterozygote GC vs. GG, dominant GC + CC vs. GG, and recessive CC vs. GG + GC (all *P*-value of association test > 0.05). Further, a total of 465 OPMD patients and 775 oral squamous cell carcinoma (OSCC) ones were identified from 8 eligible case–control studies on this polymorphism in OPMD progression to OSCC. The analyses revealed that there was also no significantly risk of OPMD progression in the cases for the genetic models (all *P*-value of association test > 0.05).

**Conclusion:**

Our data of a pooled-analysis indicates that TP53 codon 72 polymorphism may not act as genetic factor for the risk of OPMD onset and progression. Combined with the conclusion by a systematic review and meta-analysis, we put forward a new opinion that TP53 genetic typing cloud not influence p53 protein expression in OPMD.

**Supplementary Information:**

The online version contains supplementary material available at 10.1186/s12903-023-03316-0.

## Introduction

Oral potentially malignant disorders (OPMD) contain a group of lesions such as oral leukoplakia (OLK), oral erythroplakia, oral lichen planus, and oral submucous fibrosis, which carry a significantly increased risk for malignant progression to oral squamous cell carcinoma (OSCC) [1]. The onset and progression of OPMD arise as a result of a multi-step carcinogenic process that correlates to the accumulation of genetic and epigenetic alterations [2]. Single nucleotide polymorphisms (SNP) in gene encoding for susceptibility factors may influence gene expression, protein function, and disease predisposition [3]. Although the etiology and progression of OPMD are quite complex, evidence indicates that single-nucleotide polymorphisms of candidate genes may be associated with genetic susceptibility of the disorders [4, 5].

Tumor suppressor p53 (TP53) gene is regarded as potential guardian of the human genome [6]. TP53 is the most common mutated gene in head and neck cancer, making p53 an appealing target for improving treatment of head and neck cancer by restoring its tumor suppressor action [7, 8]. The rs1042522 G/C polymorphism of TP53 results in the alteration at codon 72 between arginine (Arg) and proline (Pro) and causes the TP53Arg72Pro mutation. This may affect the normal function of the TP53 protein and is implicated in susceptibility to several cancers including OSCC [9–11]. A earlier meta-analysis published in *BMC Oral Health*reported TP53 codon 72 polymorphism was not associated with OLK susceptibility [12]. Recently, a systematic review and meta-analysis demonstrated that overexpression of p53 immunoprotein was significantly associated with the risk of OPMD malignant progression [13].

Based on the above, we hypothesize that TP53 polymorphism may be associated with the risk of OPMD progression to OSCC. Besides, there are newly published several case–control studies on various types of OPMD, which are suitable to assess the association of TP53 codon 72 polymorphism with the onset of general OPMD. Therefore, the objective of the meta-analysis was to systematically evaluate the relationship between TP53 codon 72 polymorphism and both onset and progression of OPMD based on case–control studies.

## Materials and methods

### Search strategy and data extraction

A comprehensive literature search was conducted on PubMed, Web of Science, and Medline databases for all relevant publications on the association between TP53 codon 72 polymorphism and OPMD, without any restriction on Feb. 21, 2023. According to the search strategy described in Supplementary Table S1, we used medical subject term (‘‘polymorphism*’’ OR ‘‘gene variant’’) AND (‘‘p53 OR ‘‘TP53’’) AND the synonyms of OPMD in all fields. The inclusion criteria for eligible articles were as follows: (i) human case–control studies; (ii) evaluation of TP53 codon 72 polymorphism and OPMD onset or progression. OPMD progression indicates the polymorphism in OPMD compared with in OSCC; (iii) sufficient genotyping data for the computation of odds ratio (OR) and 95% confidence interval (CI); (iv) histologically confirmed diagnosis of OPMD and OSCC. On the contrary, the exclusion criteria were as follows: (i) not a case–control study; (ii) overlapping or duplicate publications; (iii) no genotype data reported.

According to the selection criteria, all relevant crude data were extracted from each eligible study independently by two investigators. Inconsistency was discussed until a consensus was obtained with a third investigator. The following information were extracted from each study: first author' name, publication year, country origin, ethnicity, age, sex, tobacco and alcohol use, genotyping methods, number and characteristics of cases and controls, genotype distributions of cases and controls. The case group was OPMD, and control group was healthy individuals or OSCC. Ethical approval and informed consent were not applicable for a meta-analysis. Base on the above process and the flow diagram of the Preferred Reporting Items for Systematic Reviews and Meta-Analyses (PRISMA) guideline (Supplementary Figure S1), 13 eligible case–control studies on TP53 codon 72 polymorphism and OPMD onset or progression were retrieved for detailed evaluation from the literature databases (Table 1) [14–26].

**Table 1:** The characteristics of the included case–control studies on p53 protein codon 72 polymorphism in onset and progression of OPMD

Author, Year	Country	Ethnicity	Study design	Mean age (y)	Male (%)	Tobacco (%)	Alcohol (%)	Genotyping method	HWE (*P* value)
Mitra et al. 2005 [14]; Misra et al. 2009 [15]	India	Asian	342 HC^a^	50.4	76.1	53.4	46.5	PCR–RFLP	0.444
			191 OLK	47	86.3	96.5	15.7		
			308 OSCC	55	63.5	43.2	18.4		
Lin et al. 2008 [16]	China	Asian	280 HC	52.1	47.5	15.7	21.1	PCR–RFLP	0.328
			39 OSF	43.1	100	94.9	59		
			70 OLK	49.8	92.9	81.4	82.9		
			297 OSCC	49.5	92.6	81.5	68.4		
Ye et al. 2008 [17]	USA	Mixed	137 HC	59.1	55.8	44.9	68	PCR–RFLP	0.532
			110 OLK	57.4	55.8	68.7	53.7		
Ghabanchi et al. 2009 [18]	Iran	Asian	93 HC	46.7	43	NA	NA	PCR-SSP	0.001
			25 OLP	43.1	44	NA	NA		
Yanatatsaneeji et al. 2010 [19]	Thailand	Asian	94 HC	33.6	32.6	NA	NA	PCR–RFLP	0.999
			97 OLP	36.2	53.2	NA	NA		
Sikka et al. 2014 [20]	India	Asian	98 HC	49	87	59.3	NA	PCR–RFLP	0.479
			86 OLK	48	90.1	45	NA		
Zarate et al. 2017 [21]	Argentina	Mixed	18 HC	NA	50	NA	NA	PCR–RFLP	0.139
			30 OLP	NA	33	NA	NA		
			14 OLK	NA		NA	NA		
			44 OSCC	NA	63	NA	NA		
Ramya et al. 2017 [22]	India	Asian	25 HC	NA	NA	36	NA	PCR–RFLP	0.982
			15 OLK	46.7	80	60	NA		
Tandon et al. 2017 [23]	India	Asian	21 HC	NA	NA	NA	NA	PCR–RFLP	0.879
			6 OLK	NA	82.9	NA	NA		
			35 OSCC	NA		NA	NA		
Tabatabaei et al. 2018 [24]	Iran	Asian	30 OLP	43.3	21.1	NA	NA	PCR	0.616
			20 OSCC	58.2	39.3	NA	NA		
Hallikeri et al. 2019 [25]	India	Asian	30 HC	NA	86.7	NA	NA	PCR	0.219
			30 OSF	NA	100	NA	NA		
			30 OSCC	NA	86.7	NA	NA		
Galíndez et al. 2021 [26]	Argentina	Mixed	35 HC	NA	39.5	21.1	31.6	PCR–RFLP	0.953
			55 OLK/OLP	NA	37.7	73.8	31.1		
			41 OSCC	NA	65.9	57.6	60.6		

*HC* health control, *HWE* Hardy–Weinberg equilibrium, *OLK* oral leukoplakia, *OLP* oral lichen planus, *OPMD* oral potentially malignant disorders, *OSCC* oral squamous cell carcinoma, *OSF* oral submucous fibrosis, *PCR* polymerase chain reaction, *PCR–RFLP* PCR-restriction fragment length polymorphism, *PCR-SSP* PCR-single-specific primer

^a^The 2 studies used the same health control group (*n* = 342)

### Statistical analysis

As per the methods described previously [27], the statistical analysis was carried out with the software Review manager 5.4 (The Cochrane Collaboration, Oxford, UK). Hardy–Weinberg equilibrium (HWE) of control group in included studies was measured using Pearson’s goodness-of-fit χ^2^ test. The strength of association of TP53 codon 72 polymorphism and OPMD onset or progression was determined by calculating odds ratios (ORs) with corresponding 95% credible interval (CI). χ^2^–based Q-test and I^2^ statistics were utilized to test statistical heterogeneity, and the Z-test was used to assess the statistical significance of the pooled OR. ORs were pooled according to the fixed-effects model (Mantel–Haenszel model) if heterogeneity was not significant (*P* > 0.05). Otherwise, the random-effects model (DerSimonian and Laird model) was conducted. Begg's funnel plot and Egger's test were visually examined to evaluate the potential publication bias of the included studies. All tests were used two-sided *P* value, and the value less than 0.05 was accepted as statistical significance.

## Results

### Association of TP53 codon 72 polymorphism with OPMD onset

**Fig. 1:**
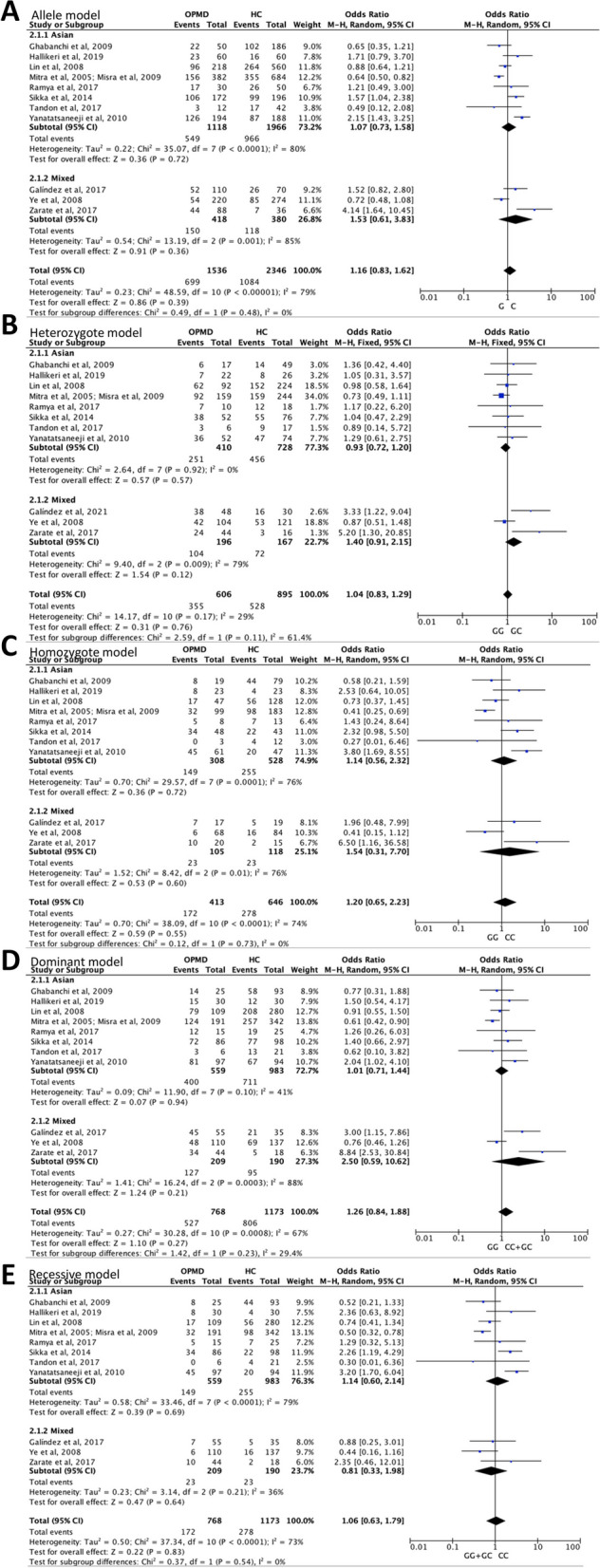
Detailed genotype distributions and forest plots of TP53 codon 72 polymorphism with OPMD onset compared to healthy control (HC) in five genetic models. **A** allele C vs. G, **B** heterozygote GC vs. GG, **C** homozygote CC vs. GG, **D** dominant GC + CC vs. GG, **E** recessive CC vs. GG + GC

There were 12 eligible case–control studies on TP53 codon 72 polymorphism in OPMD onset, compare to health control (Table 1). A total of 768 OPMD patients and 1173 healthy individuals were identified from six countries including India, China, USA, Argentina, Iran, and Thailand. In the overall analysis, no significantly increased or decreased risk of OPMD onset was observed in the cases for the five genetic models including allele C vs. G [*P*_A_ (P-value of association test) = 0.39], homozygote CC vs. GG (*P*_A_ = 0.55), heterozygote GC vs. GG (*P*_A_ = 0.76), dominant GC + CC vs. GG (*P*_A_ = 0.27), and recessive CC vs. GG + GC (*P*_A_ = 0.83). In the stratified analysis by ethnicity, the similar results were observed in five genotype models among Asians and mixed ethnicity. The detailed genotype distributions and forest plots of the five genetic models are depicted in Fig. 1. Begg’s funnel plot showed that there was no obvious evidence for publication bias in five genetic models of TP53 codon 72 polymorphism in OPMD onset (Figure S2). These results indicate that TP53 codon 72 polymorphism may have no significant influence on the risk of OPMD onset.

### Association of TP53 codon 72 polymorphism with OPMD progression

**Fig. 2:**
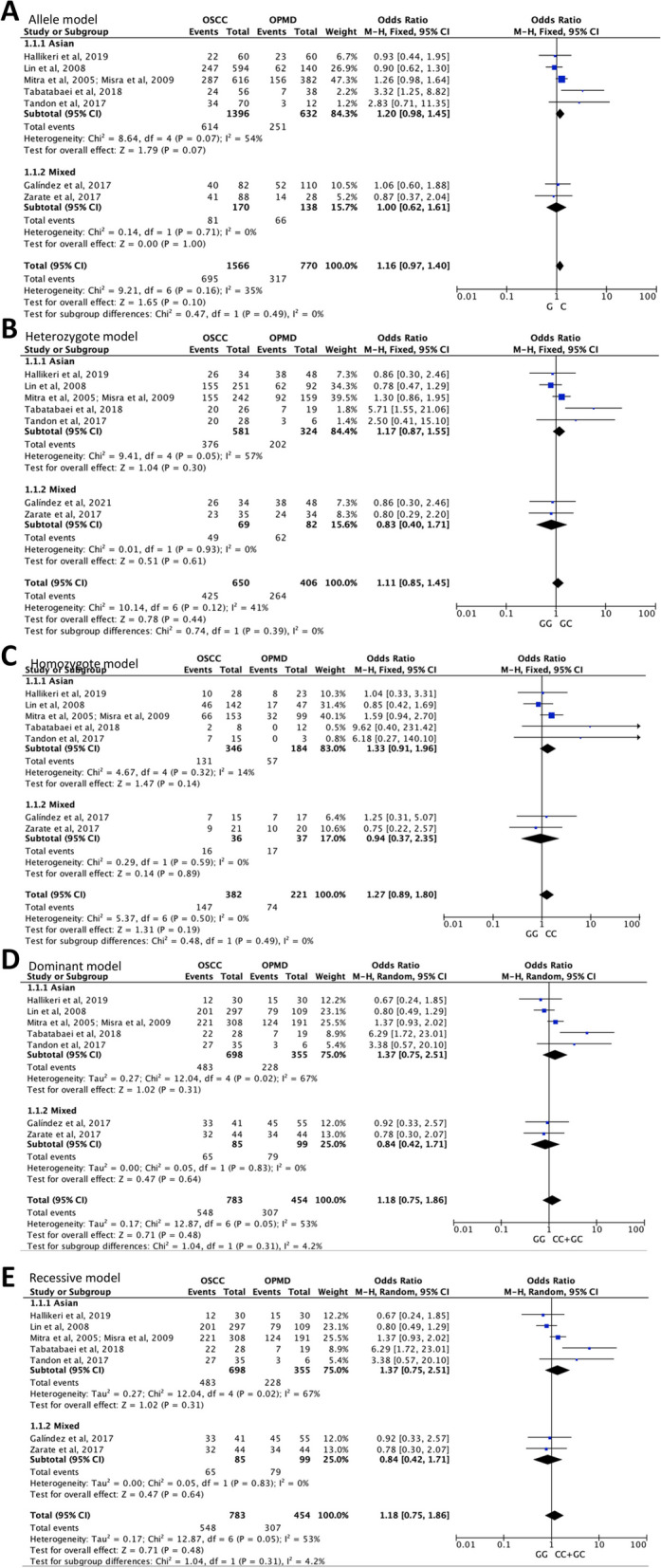
Detailed genotype distributions and forest plots of TP53 codon 72 polymorphism with OPMD progression to OSCC in five genetic models. **A** allele C vs. G, **B** heterozygote GC vs. GG, **C** homozygote CC vs. GG, **D** dominant GC + CC vs. GG, **E** recessive CC vs. GG + GC

There were 8 eligible case–control studies on TP53 codon 72 polymorphism in OPMD progression to OSCC (Table 1). A total of 465 OPMD patients and 775 OSCC ones were identified from four countries including India, China, Argentina, and Iran. In the overall analysis, no significantly increased or decreased risk of OPMD progression to OSCC was observed in the cases for the five genetic models including allele C vs. G [*P*_A_ = 0.10], homozygote CC vs. GG (*P*_A_ = 0.19), heterozygote GC vs. GG (P_A_ = 0.44), dominant GC + CC vs. GG (*P*_A_ = 0.48), and recessive CC vs. GG + GC (*P*_A_ = 0.48). In the stratified analysis by ethnicity, the similar results were observed in four genotype models among Asians and mixed ethnicity. Constrainedly, an association of TP53 codon 72 polymorphism in allele model with OPMD progression was found (OR, 1.20; 95%CI, 0.98–1.45; *P* = 0.069) among Asians based on 6 studies. The detailed genotype distributions and forest plots of the five genetic models are illustrated in Fig. 2. Begg’s funnel plot showed that there was no obvious evidence for publication bias in five genetic models of TP53 codon 72 polymorphism in OPMD progression (Figure S3). These results indicate that TP53 codon 72 polymorphism may have no significant influence on the risk of OPMD progression to OSCC.

## Discussion

Given the fact that TP53 exhibits diverse behaviors and is involved in various regulatory roles during carcinogenesis, elucidating the role of wild-type and mutated TP53 in the development of OSCC remains a challenge [5–8]. In an updated meta-analysis, Sun et al. [12] conducted an updated meta-analysis of 17 case–control studies from 16 articles with 3047 cases of OSCC and 3305 health controls, and concluded that there was no significant association between TP53 codon 72 polymorphism and the risk of OSCC in either the Asian or Caucasian population. This data was in agreement with the result from another contemporaneous meta-analysis [28]. Interestingly, Sun et al. [12] performed an additional meta-analysis of 6 case–control studies from 5 articles with 391 cases of OLK and 763 health controls, and also found that no significant association of TP53 codon 72 polymorphism with OLK susceptibility. In this study, we highlighted the potential role of TP53 codon 72 polymorphism in the risk of onset and progression of general OPMD (containing OLK, oral lichen planus, and oral submucous fibrosis) through a pooled-analysis of 13 case–control studies. Overall, the results of this study indicated that TP53 codon 72 polymorphism may not also be associated with the risk of OPMD onset and progression.

Recently, overexpression of p53 immunoprotein was demonstrated to be significantly associated with malignant progression of OPMD [13]. This was inconsistent with the result of TP53 codon 72 polymorphism not associated with OPMD progression in this study, suggesting p53 overexpression in OPMD progression could be not influenced by TP53 codon 72 polymorphism. Tandon et al. examined TP53 codon 72 gene polymorphism and p53 immunoexpression in 6 cases of OLK and 35 cases of OSCC, but they did not investigate the relationship between TP53 polymorphism and p53 immunoexpression in two groups. Zhang et al. [29] reported that p53 protein expression was identified to be affected by TP53 codon 72 polymorphism in low rectal cancer. Dastjerdi MN [30] reported that TP53 codon 72 polymorphism may be correlated with p53 overexpression and increased risk for colorectal cancer. Al-Dhaheri et al. [31] reported that p53 overexpression in the progression towards malignancy of preneoplastic and neoplastic rat mammary glands associated with TP53 polymorphism; while Rybárová et al. [32] reported that no statistically significant difference was found between TP53 codon 72 polymorphism and p53 protein expression in human breast cancer. These variations in results might be possible due to organ specificity and species differences.

Meta-analysis allows stronger quantitative synthesis for identifying some models of risk markers, and reduces the limitations of the relatively small sample size and sampling bias of individual studies [12, 28]. Although the efforts in performing a comprehensive analysis, certain limitations need to be addressed in this study. First, the number of eligible studies available with the pooled sample size of most studies was small and in both overall and subgroup analyses; and it is possible that some relevant studies in some localized databases were missed. Secondly, the effect of the confounding ingredients in gene-environment exposures and lifestyle habits interactions such as environmental factors, and tobacco and alcohol use, were not estimated in the current study due to data available limitation (Table 1). Thirdly, the results were of heterogeneity in some genetic models, possible due to role of various factors such as geographic distribution and racial differences on various predisposing factors involving lifestyle habits. Therefore, to obtain a more accurate results of TP53 codon 72 polymorphism on OPMD onset and progression, additional well-designed studies with larger sample sizes and diverse ethnicities are warranted to validate the associations.

In summary, this is the first pooled-analysis to investigate the association between TP53 codon 72 polymorphism and OPMD onset and progression, suggesting that TP53 polymorphism may not act as genetic factor for the risk of this disease. Combined with the conclusion by a systematic review and meta-analysis [13], we put forward a new opinion that TP53 genetic typing cloud not influence p53 protein expression in OPMD. Further studies are needed to consolidate this opinion.

### Supplementary Information

**Additional file 1: Table S1.** Search strategy in literature database.

**Additional file 2: Figure S1.** Flow diagram of the study selection process. **Figure S2.** Begg’s Funnel plots of association between TP53 codon 72 polymorphism with OPMD onset in (A) allele model, (B) heterozygote model, (C) homozygote model, (D) dominant model, (E) recessive model. **Figure S3.** Begg’s Funnel plots of association between TP53 codon 72 polymorphism with OPMD progression in (A) allele model, (B) heterozygote model, (C) homozygote model, (D) dominant model, (E) recessive model.

## Data Availability

All data generated or analyzed during the present study are included in this published article.
