# Supplementary material for: Systematic evaluation of TP53 codon 72 polymorphism associated with onset and progression of oral potentially malignant disorders
Source: BMC Oral Health. 2023 Sep 12;23:659. doi: 10.1186/s12903-023-03316-0 (PMC10496165; doi:10.1186/s12903-023-03316-0)
Supplement: Supplementary file 1 — Additional file 1: Table S1. Search strategy in literature database. [file 12903_2023_3316_MOESM1_ESM.docx]

**Table S1. Search strategy in literature database**

| Search strategy |
| --- |
| (p53 OR TP53) AND oral [title] AND (polymorphism OR gene variant) AND (potentially malignant [Title/Abstract] OR OPMD OR leukoplakia OR erythroplakia OR dysplasia OR dysplastic OR preneoplastic OR preneoplasia OR precancerous OR precancer OR premalignant OR premalignancy OR intraepithelial neoplasia OR lichen planus OR lichenoid OR actinic cheilitis OR lupus erythematosus OR submucous fibrosis) |
