# Supplementary material for: Systematic evaluation of TP53 codon 72 polymorphism associated with onset and progression of oral potentially malignant disorders
Source: BMC Oral Health. 2023 Sep 12;23:659. doi: 10.1186/s12903-023-03316-0 (PMC10496165; doi:10.1186/s12903-023-03316-0)
Supplement: Supplementary file 2 — Additional file 2: Figure S1. Flow diagram of the study selection process. Figure S2. Begg’s Funnel plots of association between TP53 codon 72 polymorphism with OPMD onset in (A) allele model, (B) heterozygote model, (C) homozygote model, (D) dominant model, (E) recessive model. Figure S3. Begg’s Funnel plots of association between TP53 codon 72 polymorphism with OPMD progression in (A) allele model, (B) heterozygote model, (C) homozygote model, (D) dominant model, (E) recessive model. [file 12903_2023_3316_MOESM2_ESM.pdf]

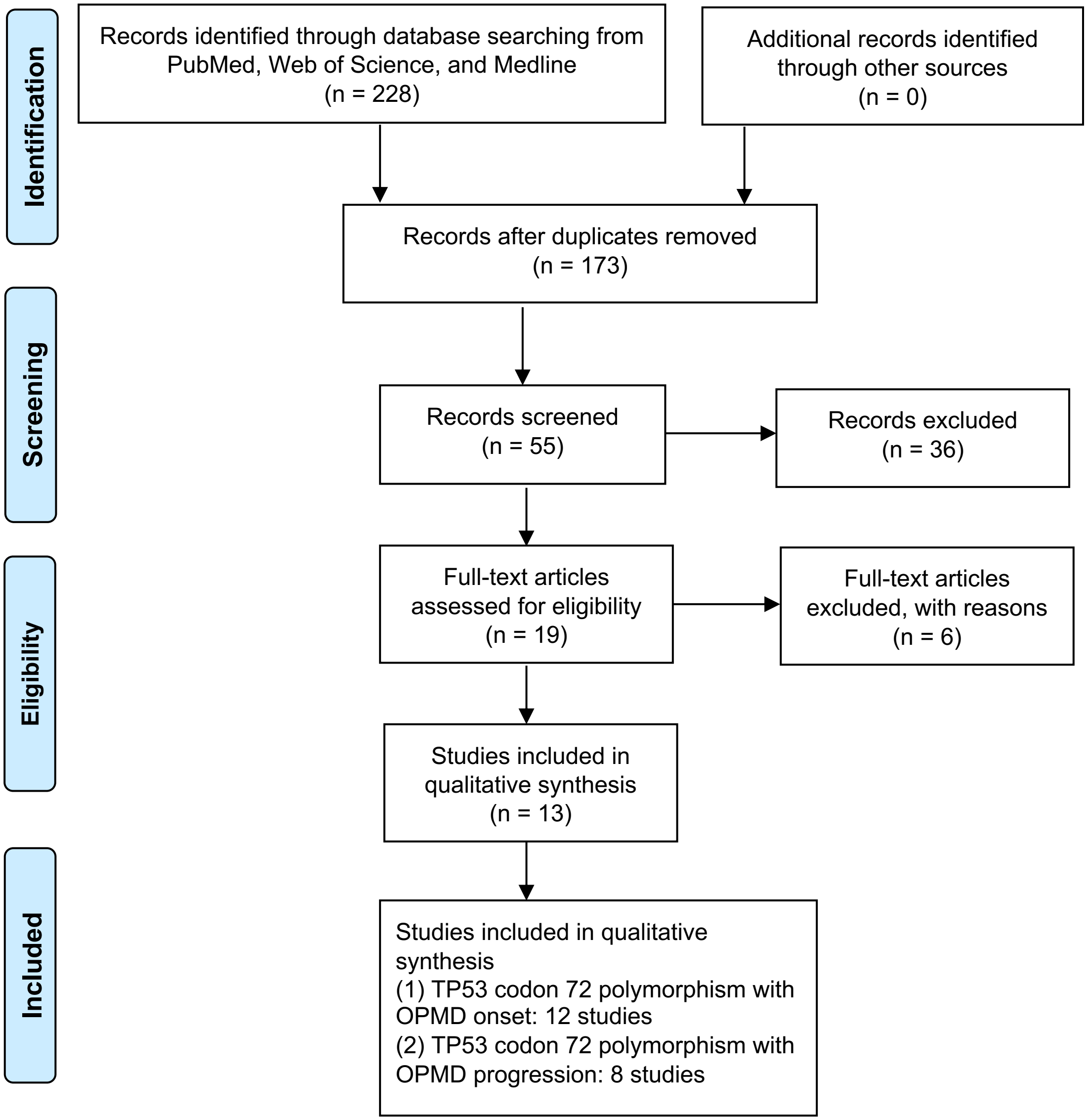

Figure S1. Flow diagram of the study selection process.

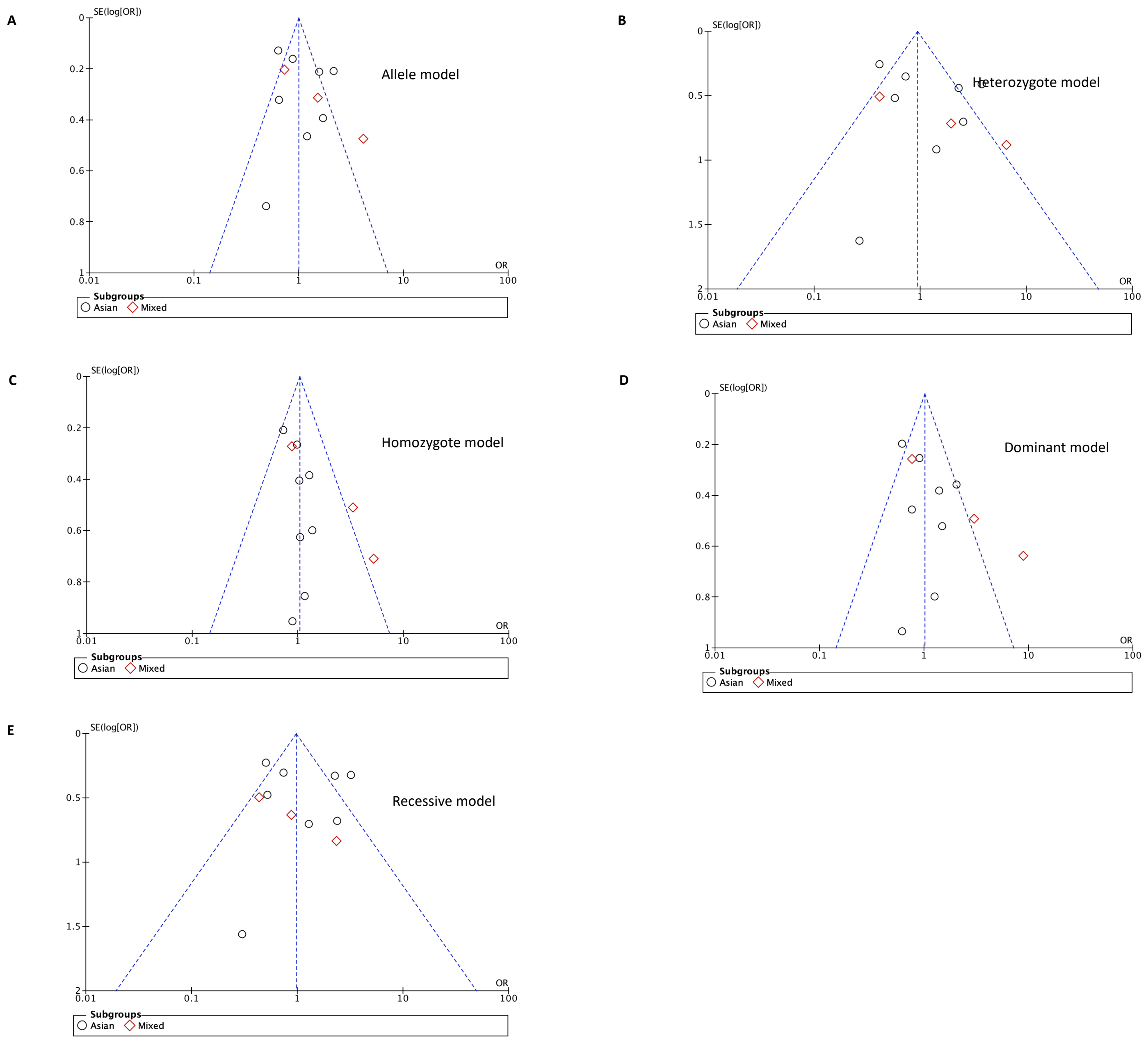

Figure S2. Begg's Funnel plots of association between TP53 codon 72 polymorphism with OPMD onset in (A) allele model, (B) heterozygote model, (C) homozygote model, (D) dominant model, (E) recessive model.

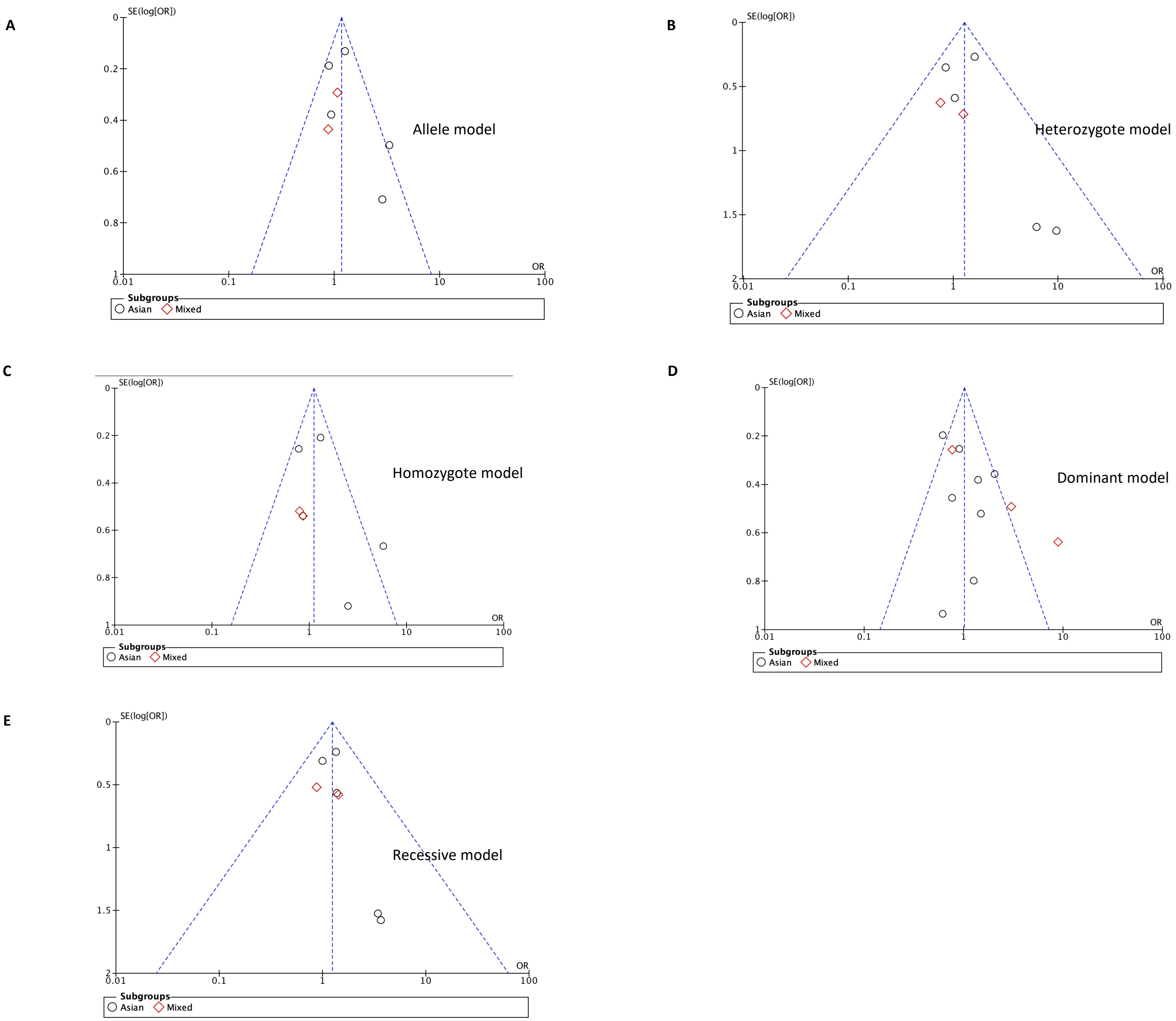

Figure S3. Begg's Funnel plots of association between TP53 codon 72 polymorphism with OPMD progression in (A) allele model, (B) heterozygote model, (C) homozygote model, (D) dominant model, (E) recessive model.
